# Optimized two-stage time-truncated control chart for Weibull distribution

**DOI:** 10.1038/s41598-024-52619-x

**Published:** 2024-01-24

**Authors:** Shafaqat Ali, Jose Jorge, Muhammad Aslam, Muhammad Kashif

**Affiliations:** 1 School of Mathematical Sciences, Universiti Sains Malaysia, 11800 Minden, Penang Malaysia; 2 Department of Mathematics, National University of Modern Languages, H-9, Islamabad, Pakistan; 3 School of Applied Science and Engineering, Universidad EAFIT, 050022 Medellín, Colombia; 4 Department of Statistics, Faculty of Science, King Abdulaziz University, 21551 Jeddah, Saudi Arabia; 5 Department of Mathematics and Statistics, Faculty of Sciences, University of Agriculture, Faisalabad, Pakistan

**Keywords:** Engineering, Mathematics and computing

## Abstract

In this article, an attribute control chart is proposed when the lifetime of a product follows a Weibull distribution in two-stage sampling, which is based on the number of failures from a truncated life test. The coefficients of the proposed double sampling attribute control chart and the test duration are determined so that the average run length when the process is in control is close to the target value. An overview is reported on how double sampling np control charts work. Tables reporting the out-of-control average run lengths are given for various shift parameters. A case study is given to illustrate the proposed control chart for industrial use. A comparison of two-stage and single-stage sampling of failure of products is discussed.

## Introduction

In manufacturing industries, control charts can be used to monitor the quality of high-quality product production. These tools assist in ensuring that items are manufactured within specified limits by monitoring quality in advance. Many control charts have been developed to monitor processes and address real-life production issues in the industrial sector. Usually, a normal distribution is used for quality characteristics. However, in some instances, non-normal distributions are employed when quality characteristics do not follow a normal distribution. Therefore, control charts constructed under the assumption that the quality characteristic follows a normal distribution may mislead the experimenter if the quality does not follow a normal distribution. In our research, we incorporated the concept of double-stage sampling, which refers to a technique within control charts involving two distinct stages. The first stage involves extracting a sample from the population, with the sample size determined at this point. In the second stage, the sample is subdivided into subgroups, and a control chart is created based on the data from these subgroups. This method is used to reduce the variability of the data and to improve the accuracy of the control chart. Moreover, DS-np control charts are an important tool for monitoring process performance and detecting changes in process performance. These charts are used to identify and analyze process variation and to detect special causes of variation. DS-np control charts can help identify process problems, identify process improvement opportunities, and provide feedback to process owners and operators. They can also be used to monitor process performance over time and to detect changes in process performance. The Weibull distribution can be used both in the medical and engineering fields. Firstly, Weibull distribution is commonly used in biostatistics to model survival data. It is used to analyze the time to failure of a system or the time to an event, such as death or recovery from a disease. It is also used to model the time to onset of a disease, the time to recovery from a disease, and the time to progression of a disease. Additionally, for biostatistics, Weibull distribution is used to model the time to respond to treatment. Secondly application in an engineering field, Weibull distribution is widely used in industrial engineering and quality control for a variety of purposes. It can be used to model the time to failure of a product, the time to complete a task, or the time to complete a process. It can also be used to model the probability of a product failure within a fixed time frame. It can be used to model the probability of a process or task taking longer than expected. Finally, it can be used to model the probability of a product. Below, there are various studies on control charts where the process does not satisfy the normality assumptions. Weibull distribution is widely used for reliability and quality engineering. A control chart for positively skewed distribution like Weibull, gamma, and log-normal has been discussed by Refs.^1–3^ developed a nonnormal distribution as a Gamma distribution which is considered a failure model for the economic statistical design $\overline{X }$ control charts. The parametric bootstrap method for the detection of lower and upper control limits was established by Ref.^4^ and used to monitor the process shift of the percentile of the Weibull population. Another economic-statistical design of $\overline{X }$ control charts for non-normal quality measurements with the assumption of the average sample following the Johnson distribution and sensitivity of mean shift and in control time measured using Weibull distribution by Ref.^5^. A two-plan sampling system proposed by Ref.^6^ for failure-censored life testing when lifetime follows Weibull distribution. They^7^ suggested a control chart based on failure-censored reliability tests with the assumption that the sample follows to Weibull distribution. A non-normal approach was developed by Ref.^8^ for the observation of control chart features where data follow a non-normal distribution as a generalized exponential distribution. A real-life application of lifetime data based on conditional mean and median based on cumulative sum control charts developed by Ref.^9^. Two lifetime distributions are discussed named Transmuted Power function distribution and survival weighted Power function distribution discussed by Ref.^10^ for performance measure of attribute control charts. The coefficients of control limits for various sample sizes and truncation coefficients depend on the target ARL value and shift coefficient explored and computed using simulation and real-life examples from Refs.^10,11^ explored Shewhart control charts which are used to monitor the Weibull mean based on the gamma distribution. Another control chart was proposed under the assumption of Weibull distribution by Ref.^12^. In this article, we will extend a single sampling extension as double sampling for the attribute control chart using a truncated life test completely discussed in “Design of the control charts” section. This paper considered discussing the double sampling attribute chart for the Weibull distribution. An optimization model for average run length in control (IC) and out-of-control (OOC) was discussed. A comparison of single sampling and double sampling is also reported for attribute inspection for the ${\text{np}}$ control chart. The use of Weibull distribution under the truncated life test for DS can be used to handle the lifetime of attribute control charts. The Weibull distribution can be used to calculate the probability of the control chart failing within a certain period, which can be used to determine the lifetime of the control chart, the importance of this distribution discussed by Ref.^2^.

Upon delving into the existing body of literature, substantial research has been conducted on crafting control charts for instances where counts originate from truncated tests. Despite an extensive review of the literature, it has come to our attention that no prior work has been undertaken to devise an optimized two-stage control chart utilizing the Weibull distribution. This paper aims to fill this gap by introducing the design of an optimized two-stage control chart specifically tailored for situations where counts are recorded from truncated life tests. We will elucidate the methodology for determining control chart parameters within predefined optimization constraints. Additionally, a simulation study will be presented, along with the application of the proposed control chart using a real-world example. Our analysis will include a comparative evaluation, demonstrating the efficiency of the proposed control chart over an existing counterpart introduced by Ref.^13^. We anticipate that our proposed control chart will outperform the existing one in terms of average run length.

## Design of the control charts

Suppose that the failure time of a product follows a Weibull distribution whose cumulative distribution function (cdf) is given by

1 $${\text{F}}\left({\text{t}};\upbeta ,\uptheta \right)=1-{{\text{exp}}\left(-\frac{{\text{t}}}{\upbeta }\right)}^{\uptheta },\mathrm{ t}\ge 0.$$

In Eq. (1), $\theta$ is the known shape parameter and $\beta$ is the unknown scale parameter. The shape parameter is known on the engineering experience discussed by Refs.^6,14^. The application of mean time failure for single-stage sampling for attribute charts was discussed by Ref.^15^. The expected value of the product’s mean life is expressed using a probability distribution, as represented in Eq. (2).

2 $${\text{E}}\left({\text{X}}\right)=\upmu =\left(\frac{\upbeta }{\uptheta }\right)\Gamma \left(\frac{1}{\uptheta }\right),$$

where $\Gamma$ is the gamma function. Here we will compute the mean of an attribute two-stage sampling control chart for monitoring the sample observation $n={n}_{1}+{n}_{2}$ for the mean shift by deducting the number of failed items of some specified ${t}_{0}$ (Truncated time). Let ${\mu }_{0}$ be the target mean life when the process IC and ${\mu }_{1}$ will be used, which indicates the shifted process is OOC. The probability that an item fails by time ${t}_{0}$ is given as

3 $$p=1-{\text{exp}}\left({\left(-\frac{{t}_{0}}{\beta }\right)}^{\theta }\right).$$

If we specify the truncated time ${t}_{0}$ in terms of multiple IC, process means through ${t}_{0}=c{\mu }_{0}$ for a constant c is known as a truncated time constant. An unknown parameter $\theta$ in terms of the mean from Eq. (1) and then Eq. (3) can be written as

4 $${\text{p}} = 1 - \exp \left( { - {\text{c}}^{{\uptheta }} \left( {{ }\frac{{\upmu }}{{\upmu }}_{0} } \right)^{{ - {\uptheta }}} } \right)\left( {\frac{{\bigg\lceil {\frac{1}{{\uptheta }}} }}{{\uptheta }}} \right)^{{\uptheta }} { }.$$

If the process is IC (that is, $\mu ={\mu }_{0}$), then the probability in Eq. (4), reduces to

5 $$p_{0} = 1 - \exp \left( { - c^{\theta } \left( {\frac{{\bigg\lceil {\frac{1}{\theta }}}}{\theta }} \right)^{\theta } } \right).$$

A new double-sampling control chart for measuring the failure lifetime of a product using Weibull distribution is the extension of the single sampling control chart proposed by Aslam and Jun^13^. The scheme of double sampling np control chart for failure lifetime can be assessed through the failure of product combined on second stage sample which comprises the five parameters as: ${n}_{1}, {n}_{2}, WL, UC{L}_{1} \,and\, UC{L}_{2}$ proposed by Ref.^16^. Steps involved in measurement scheme for the failure of products under second stage sampling which follows the Weibull distribution.

### Step 1

Begin with attribute inspection on inspection level 1st and the sample number represented by ${n}_{1}$ (count the number of failure items of the specification time ${t}_{0}=c{\mu }_{0}$ where ${\mu }_{0}$ is the target mean when the process is IC and $c$ is any constant).

### Step 2

The process will be considered OOC on 1st stage sampling if ${d}_{1}<WL$ or ${d}_{1}>UC{L}_{1}$ and IC if $LCL\le {d}_{1}\le UC{L}_{1}$ according to Ref.^14^.

### Step 3

An additional sample is required in case of $WL<{d}_{1}<UC{L}_{1}$ according to De Araujo Rodrigues et al.^16^. In the second stage of sampling defective items counted as $D={d}_{1}+{d}_{2}$. The newly proposed IC and OOC scenario will be as a DS-np chart: if $D<UC{L}_{2}$ and OOC for $D>UC{L}_{2}$. So the random variable $D$ follows the binomial distribution with parameter $n$ and ${p}_{0}$ when the process is IC where ${p}_{0}$ is the probability an item fails on first or second-stage sampling. In the present study, our main focus is to measure the lifetime of a product when a second sample has occurred. So during an inspection in the second stage of sampling our main focus with total defective items $D$ occurred on the inspection level second.

## Scheme and algorithm of control chart for the failure of products

We have defined the following control limits: $WL, UC{L}_{1}$ and $UC{L}_{2}$. For a better representation of the schemes, we can add or subtract a decimal from the floor or ceiling of these real values:

$${\text{WL}} = \left\lfloor {{\text{WL}}} \right\rfloor + 0.5,$$

$${\text{UCL}}_{1} = \left\lceil {{\text{UCL}}_{1} } \right\rceil - 0.5,$$

$${\text{UCL}}_{2} = \left\lfloor {{\text{UCL}}_{2} } \right\rfloor + 0.5.$$

**Figure 1:**
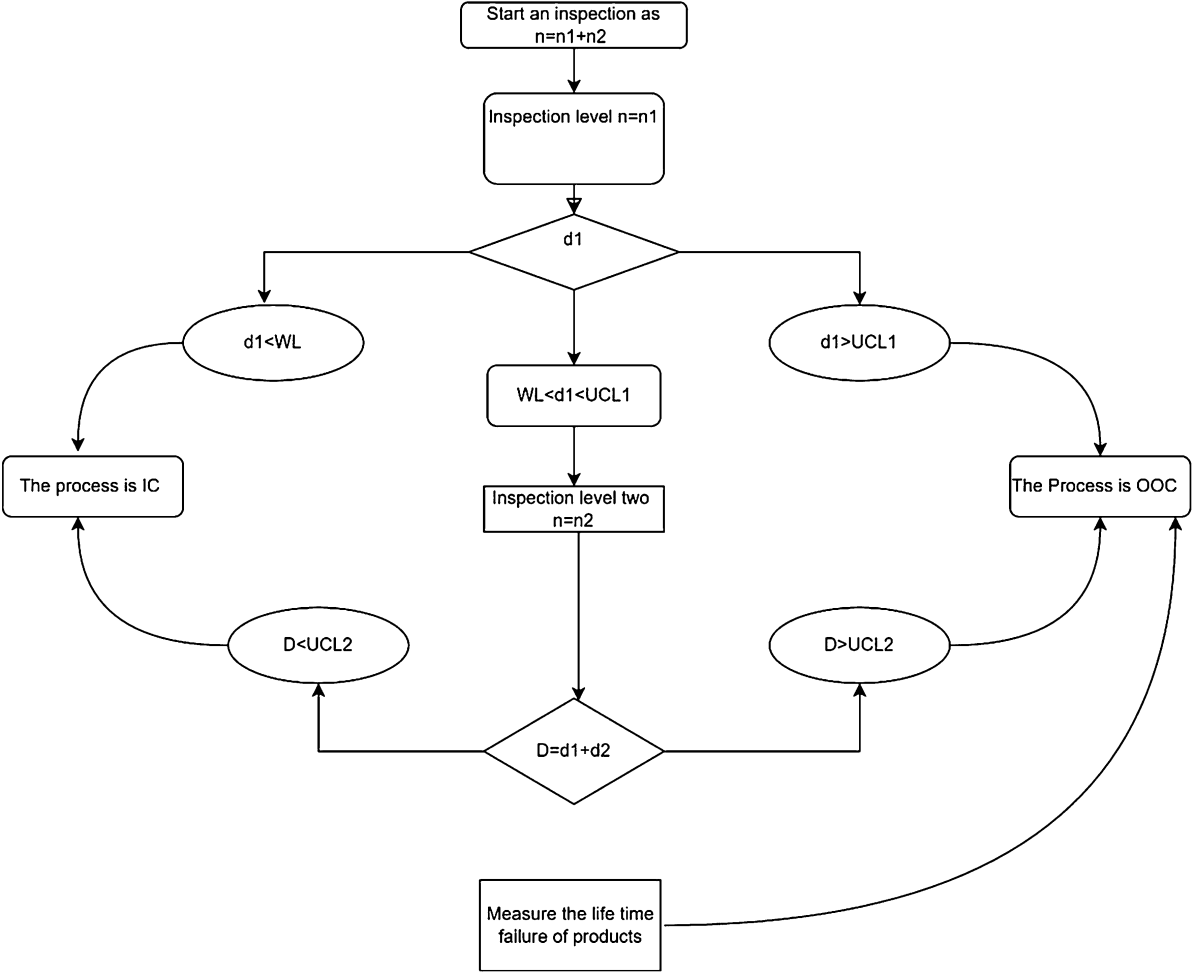
Time failure of products during attribute inspection at the second stage.

For the Double-sampling attribute control chart, a flow chart Fig. 1 is given below which is a sketch used to monitor and control a process. It is used to detect and identify any special causes of variation in the process. Here based on the prospered control chart scheme, the double sampling attribute flow chart consists of two samples as $n={n}_{1}+{n}_{2}$. In Fig. 1 the complete sequence of taking sub-sample and declaring whether an item is defective or not and process is OOC or IC is discussed.

In this study we will consider process IC for both stage sampling and the probability of IC can be used as:

6 $$P_{{n_{1} }} = {\text{Pr}}\left( {d_{1} \le \left\lfloor {WL} \right\rfloor } \right) = \mathop \sum \limits_{{d_{1} = 0}}^{{\left\lfloor {WL} \right\rfloor }} \left( {\begin{array}{*{20}c} {n_{1} } \\ {d_{1} } \\ \end{array} } \right)p^{{d_{1} }} \left( {1 - p} \right)^{{n_{1} - d_{1} }} ,$$

$$P_{{n_{2} }} = \Pr \left( {\left\lfloor {WL_{1} } \right\rfloor < d_{1} < \left\lceil {UCL_{1} } \right\rceil ,d_{1} + d_{2} \le \left\lfloor {UCL_{2} } \right\rfloor } \right),$$

7 $$P_{{n_{2} }} = \mathop \sum \limits_{{d_{1} = \left\lfloor {WL} \right\rfloor + 1}}^{{\left\lceil {UCL_{1} } \right\rceil - 1}} \left[ {\left( {\begin{array}{*{20}c} {n_{1} } \\ {d_{1} } \\ \end{array} } \right)p^{{d_{1} }} \left( {1 - p} \right)^{{n_{1} - d_{1} }} \left( {\mathop \sum \limits_{{d_{2 = 0} }}^{{\left\lfloor {UCL_{2} } \right\rfloor - d_{1} }} \left( {\begin{array}{*{20}c} {n_{2} } \\ {d_{2} } \\ \end{array} } \right)p^{{d_{2} }} \left( {1 - p} \right)^{{n_{2} - d_{2} }} } \right)} \right].$$

${P}_{{n}_{1}}$ = probability of declaring the process as IC at inspection level 1. ${P}_{{n}_{2}}$ = probability of declaring the process as IC at inspection level 2.

$${P}_{0}={P}_{{n}_{1}}+{P}_{{n}_{2}} | p={p}_{0}.$$

It should be noted that if in sampling stage one ${d}_{1}=0$ If $LC{L}_{1}=0$ and on sampling stage two $LC{L}_{2}$ can not be zero. $P$ is considered IC and OOC with $p={p}_{0}$ and $p={p}_{1}$ respectively. This study focuses on two-stage sampling approach to assess the IC status of a manufacturing process. In the first stage, the probability ${{P}_{n}}_{1}$ evaluates the probability of declaring the process IC, considering potential defects ${d}_{1}$. The second stage introduces ${{P}_{n}}_{2}$, representing the probability of declaring IC while accounting for both defective units $({d}_{1}+{d}_{2})$. The combined probability ${P}_{0}$ integrates outcomes from both stages, assuming a specific probability $({p}_{0})$. Special considerations include instances with zero defects in the first stage and ensuring $LC{L}_{2}$ is non-zero in the second stage. Differentiating between IC and OOC states relies on distinct probabilities $({p}_{0}\text{ and }{p}_{1})$, offering a comprehensive evaluation of the process’s IC behavior.

Suppose now that the process mean is shifted ${\mu }_{0}$ to ${\mu }_{1}$

8 $$p_{1} = 1 - \exp \left( { - c^{\theta } \left( {\frac{{u_{1} }}{{u_{0} }}} \right)^{ - \theta } } \right)\left( {\frac{{\bigg\lceil {\left( {\frac{1}{\theta }} \right)} }}{\theta }} \right)^{\theta } .$$

If the process mean shifted as ${\mu }_{1}=g{\mu }_{0}$ for a constant $g$, then Eq. (8) becomes as

9 $$p_{1} = 1 - \exp \left( { - c^{\theta } } \right)\left( {\frac{{\bigg\lceil {\frac{1}{\theta }}}}{g\theta }} \right)^{\theta } .$$

Now the probability of the process being declared in control when the process is out of control is as follows:

$${P}_{1}={P}_{{n}_{1}}+{P}_{{n}_{2}} | p={p}_{1}.$$

The permanence measure of two-stage attribute np control charts using Weibull distribution under truncated life test will be calculated using average run length. For IC and OOC average run length is represented respectively by $AR{L}_{0}$ and $AR{L}_{1}$

For IC

10 $$AR{L}_{0}=\frac{1}{1-{P}_{0}}.$$

For OOC

11 $$AR{L}_{1}=\frac{1}{1-{P}_{1}}.$$

The control constant $g$ and truncated time constant $c$ will be first determined according to the specified n by Ref.^14^. In the present study the optimized values of ${n}_{1}$ and ${n}_{2}$ are used from the study of Ref.^15^, for the comparison of single-stage sampling and two-stage sampling lifetime of products.

On the other hand, the ASN value is calculated as follows:

$$ASN={n}_{1}+{n}_{2}\left(1-{P}_{a}\right).$$

${P}_{a}$ represents the probability associated with the control chart’s decision regarding the first sample, ${n}_{1}$.

$$P_{a} = \Pr \left( {d_{1} \le \left\lfloor {WL} \right\rfloor } \right) + \Pr \left( {d_{1} > \left\lfloor {UCL_{1} } \right\rfloor } \right),$$

$$P_{a} = \mathop \sum \limits_{{d_{1} = 0}}^{{\left\lfloor {WL} \right\rfloor }} \left( {\begin{array}{*{20}c} {n_{1} } \\ {d_{1} } \\ \end{array} } \right)p_{0}^{{d_{1} }} \left( {1 - p_{0} } \right)^{{n_{1} - d_{1} }} + 1 - \mathop \sum \limits_{{d_{1} = 0}}^{{\left\lfloor {UCL_{1} } \right\rfloor }} \left( {\begin{array}{*{20}c} {n_{1} } \\ {d_{1} } \\ \end{array} } \right)p_{0}^{{d_{1} }} \left( {1 - p_{0} } \right)^{{n_{1} - d_{1} }} .$$

## Optimization model

In Section Four, our main goal is to create a strong and effective statistical quality control chart. We do this by fine-tuning various aspects using an optimization model. The chart is designed to do a better job at catching problems in the manufacturing process, making it more reliable. Our specific focus is on reducing the time it takes to detect issues $AR{L}_{1}$ and keeping the number of samples in check (ASN) to make the whole process smoother which is prposped by Ref.^16^. We will employ a bi-objective optimization model to minimize both the $AR{L}_{1}$ and $ASN$ values. Consequently, the objective function and decision variables will be as follows:

$$\mathrm{Minimize\, AR}{{\text{L}}}_{1},\,\mathrm{ ASN},$$

$$\mathrm{Decision\, variables}:\mathrm{ WL},\mathrm{ UC}{{\text{L}}}_{1},\mathrm{ UC}{{\text{L}}}_{2},{{\text{n}}}_{1}, {{\text{n}}}_{2}.$$

The proposed control chart is subjected to the following restrictions based on DS-np ^15^:

$$0.5n<{n}_{1}<0.8n,$$

$${{n}_{1}<n}_{2}<5{n}_{1},$$

$$0 \le \left\lfloor {{\text{WL}}} \right\rfloor \le \left\lfloor {{\text{UCL}}} \right\rfloor ,$$

$$\left\lfloor {{\text{WL}}} \right\rfloor + 1 \le \left\lceil {{\text{UCL}}_{1} } \right\rceil \le \left\lfloor {{\text{WL}}} \right\rfloor + \left\lfloor {{\text{UCL}}} \right\rfloor ,$$

$$\left\lceil {{\text{UCL}}_{1} } \right\rceil + 1 \le \left\lfloor {{\text{UCL}}_{2} } \right\rfloor \le \left( {\left\lfloor {0.8*\left( {{\left\lceil {{\text{UCL}}_{1} } \right\rceil \sqrt {\frac{{n_{1} + n_{2} }}{{n_{3} }}} }} \right)} \right\rfloor - 1} \right),$$

$${\text{ASN}}\le {\text{n}},$$

$${{\text{ARL}}}_{0}\ge {{\text{r}}}_{0},$$

where ${\text{UCL}}$ is the upper control limit from a single sample Weibull chart, ${\text{n}}$ is the sample size, and ${{\text{r}}}_{0}$ is the ${{\text{ARL}}}_{0}$ value of this chart also. $\left\lfloor \cdot \right\rfloor$ denotes the “floor” of its argument, i.e. the largest integer less than or equal to the argument. $\left\lceil \cdot \right\rceil$ denotes the “ceiling” of its argument, i.e. the smallest integer larger than or equal to its argument.

## Result and discussion

In this section, we explore the practical implementation of our research findings, particularly focusing on the utilization of R software with the MCO package developed by Ref.^17^ to solve this bi-objective optimization model. Recently, Refs.^18,19^ used the same package to find optimal control limits and sample sizes of similar proposed control charts.

Below are Tables 1, 2, 3 and 4 which show the performance of the proposed control chart concerning the SS-Weibull chart through the values of the ${{\text{ARL}}}_{1}$. All the tables are presented for $\beta =1$.

**Table 1:** $AR{L}_{1}$ values for SS and DS-Weibull charts when $n=20$ and ${r}_{0} = 200$.

Shift f	SS Weibull	DS Weibull								%Gain
	${{\text{ARL}}}_{1}$	${\text{WL}}$	${{\text{UCL}}}_{1}$	${{\text{UCL}}}_{2}$	${{\text{n}}}_{1}$	${{\text{n}}}_{2}$	${\text{ASN}}$	${{\text{ARL}}}_{0}$	${{\text{ARL}}}_{1}$	
1.00	200.08	8.50	18.50	24.50	11.00	20.00	12.91	200.12	200.12	− 0.02
0.9	147.40	8.50	21.50	33.50	12.00	32.00	18.55	206.58	52.68	64.26
0.8	60.97	8.50	21.50	31.50	12.00	29.00	17.43	203.12	16.62	72.74
0.7	22.43	11.50	25.50	33.50	16.00	28.00	19.67	201.40	5.11	77.22
0.6	8.40	11.50	24.50	33.50	16.00	28.00	19.67	201.40	2.16	74.34
0.5	3.42	11.50	27.50	33.50	16.00	28.00	19.67	201.40	1.27	63.01
0.4	1.66	11.50	27.50	33.50	16.00	28.00	19.67	201.40	1.03	37.67
0.3	1.10	9.50	25.50	30.50	14.00	25.00	19.82	254.60	1.00	9.01
0.2	1.00	8.50	22.50	28.50	13.00	23.00	19.95	263.13	1.00	0.00
0.1	1.00	6.50	21.50	23.50	11.00	18.00	19.65	221.53	1.00	0.00

**Table 2:** $AR{L}_{1}$ values for SS and DS-Weibull charts when $n=20$ and ${r}_{0} = 370$.

Shift f	SS Weibull	DS Weibull								%Gain
	${{\text{ARL}}}_{1}$	${\text{WL}}$	${{\text{UCL}}}_{1}$	${{\text{UCL}}}_{2}$	${{\text{n}}}_{1}$	${{\text{n}}}_{2}$	${\text{ASN}}$	${{\text{ARL}}}_{0}$	${{\text{ARL}}}_{1}$	
1.00	370.36	9.50	17.50	20.50	11.00	19.00	11.14	370.01	370.01	0.09
0.9	197.60	10.50	25.50	33.50	15.00	33.00	17.32	384.71	94.50	52.17
0.8	75.46	8.50	22.50	34.50	13.00	36.00	18.47	429.00	24.82	67.10
0.7	27.09	8.50	22.50	34.50	13.00	36.00	18.47	429.00	7.15	73.62
0.6	9.84	8.50	21.50	34.50	13.00	36.00	18.47	429.00	2.62	73.32
0.5	3.83	8.50	21.50	34.50	13.00	36.00	18.47	429.00	1.38	63.95
0.4	1.76	7.50	20.50	33.50	12.00	35.00	19.57	475.15	1.05	40.21
0.3	1.11	8.50	23.50	28.50	14.00	25.00	19.94	405.75	1.00	9.78
0.2	1.00	7.50	22.50	25.50	13.00	21.00	19.71	457.86	1.00	0.00
0.1	1.00	6.50	20.50	23.50	12.00	19.00	19.94	408.50	1.00	0.00

**Table 3:** $AR{L}_{1}$ values for SS and DS-Weibull charts when $n=30$ and ${r}_{0} = 200$.

Shift f	SS Weibull	DS Weibull								%Gain
	${{\text{ARL}}}_{1}$	${\text{WL}}$	${{\text{UCL}}}_{1}$	${{\text{UCL}}}_{2}$	${{\text{n}}}_{1}$	${{\text{n}}}_{2}$	${\text{ASN}}$	${{\text{ARL}}}_{0}$	${{\text{ARL}}}_{1}$	
1.00	203.40	14.50	25.50	31.50	17.00	34.02	17.20	200.75	200.75	1.30
0.9	144.28	11.50	27.50	37.50	17.00	35.02	22.99	204.94	49.42	65.75
0.8	47.34	15.50	31.50	35.50	22.00	36.02	24.36	204.86	14.23	69.95
0.7	14.59	11.50	26.50	37.50	17.00	37.02	22.99	204.94	4.29	70.58
0.6	4.96	11.50	26.50	37.50	17.00	38.02	22.99	204.94	1.85	62.66
0.5	2.06	11.50	27.50	36.50	18.00	39.02	26.20	236.14	1.16	43.69
0.4	1.19	9.50	25.50	36.50	16.00	40.02	29.56	228.41	1.01	15.13
0.3	1.00	11.50	27.50	35.50	19.00	41.02	29.18	303.31	1.00	0.00
0.2	1.00	10.50	25.50	32.50	18.00	42.02	29.00	216.10	1.00	0.00
0.1	1.00	11.50	27.50	30.50	20.00	43.02	29.32	214.47	1.00	0.00

**Table 4:** $AR{L}_{1}$ values for SS and DS-Weibull charts when $n=30$ and ${r}_{0} = 370$.

Shift f	SS Weibull	DS Weibull								%Gain
	${{\text{ARL}}}_{1}$	${\text{WL}}$	${{\text{UCL}}}_{1}$	${{\text{UCL}}}_{2}$	${{\text{n}}}_{1}$	${{\text{n}}}_{2}$	${\text{ASN}}$	${{\text{ARL}}}_{0}$	${{\text{ARL}}}_{1}$	
1.00	370.05	17.50	30.50	30.50	21.00	34.02	21.09	370.16	370.16	− 0.03
0.9	244.67	14.50	29.50	36.50	20.00	35.02	21.61	375.34	86.65	64.59
0.8	74.29	13.50	28.50	37.50	20.00	36.02	23.92	387.54	20.42	72.51
0.7	21.20	10.50	25.50	37.50	16.00	37.02	22.77	383.15	5.78	72.73
0.6	6.58	12.50	28.50	37.50	19.00	38.02	24.39	372.24	2.14	67.52
0.5	2.46	10.50	26.50	37.50	16.00	39.02	22.77	383.15	1.23	50.00
0.4	1.28	9.50	25.50	36.50	16.00	40.02	27.89	449.81	1.02	20.67
0.3	1.01	12.50	28.50	36.50	20.00	41.02	27.15	450.89	1.00	0.99
0.2	1.00	12.50	28.50	34.50	21.00	42.02	29.37	420.48	1.00	0.00
0.1	1.00	11.50	27.50	32.50	20.00	43.02	29.38	403.99	1.00	0.00

The DS-Weibull chart presents better performance concerning $AR{L}_{1}$ metric for small and moderate change in the mean of the process (for values between 0.4 and 0.9 of ${\text{f}}$ approximately) compared with the SS-Weibull control chart.

In Table 5, the DS-Weibull chart demonstrates superior performance with respect to the ARL_1 metric for small and moderate changes in the mean of the process (for values between approximately 0.4 and 0.9 of f). This is in comparison to the SS-Weibull control chart, where the parameter scheme involves setting the shape parameter to 9.54 and the scale parameter to 2.055.

**Table 5:** $AR{L}_{1}$ values for SS and DS-Weibull charts when $n=25$ and ${r}_{0} = 370$.

Shift f	SS Weibull	DS Weibull								%Gain
	${{\text{ARL}}}_{1}$	${\text{WL}}$	${{\text{UCL}}}_{1}$	${{\text{UCL}}}_{2}$	${{\text{n}}}_{1}$	${{\text{n}}}_{2}$	${\text{ASN}}$	${{\text{ARL}}}_{0}$	${{\text{ARL}}}_{1}$	
1.00	370.55	7.67	14.77	17.11	12.00	50.63	20.09	369.16	370.16	00.10
0.9	246.67	0.3172	16.37	23.71	12.00	43.72	20.61	355.47	86.65	64.87
0.8	72.29	0.1498	15.25	22.50	12.50	36.91	21.92	367.51	20.42	71.75266
0.7	23.20	0.2800	15.68	17.50	12.00	66.11	21.77	383.15	5.78	75.08621
0.6	16.58	11.42	16.63	20.38	12.76	50.33	21.39	371.14	2.14	87.09288
0.5	12.46	0.3124	14.80	16.42	12.51	27.44	22.77	383.15	1.23	90.12841
0.4	1.128	5.12	14.29	21.50	12.51	36.07	22.89	389.81	1.02	9.574468
0.3	1.11	2.51	12.88	22.72	12.53	49.20	22.15	401.22	1.00	9.90991
0.2	1.10	9.96	20.92	21.72	12.74	49.77	23.37	401.48	1.00	9.090909
0.1	1.00	0.8648	10.76	20.68	12.63	56.44	24.38	413.99	1.00	0

In Table 6 Setting the Weibull distribution parameters to shape 14.54 and scale 2.69, Table 6, generated through simulation, reports improved DS-Weibull chart performance for $AR{L}_{1}$ with small to moderate mean changes (f values approximately between 0.4 and 0.9) compared to SS-Weibull control chart.

**Table 6:** $AR{L}_{1}$ values for SS and DS-Weibull charts when $n=35$ and ${r}_{0} = 370$.

Shift f	SS Weibull	DS Weibull								%Gain
	${{\text{ARL}}}_{1}$	${\text{WL}}$	${{\text{UCL}}}_{1}$	${{\text{UCL}}}_{2}$	${{\text{n}}}_{1}$	${{\text{n}}}_{2}$	${\text{ASN}}$	${{\text{ARL}}}_{0}$	${{\text{ARL}}}_{1}$	
1.00	370.55	5.21	15.96	21.85	12.50	60.32	31.04	369.16	370.88	0.02965
0.9	246.67	8.57	15.20	24.79	12.61	62.48	32.11	375.34	11.85	95.89497
0.8	72.29	6.23	20.38	24.78	12.62	48.34	33.12	387.54	20.42	71.75266
0.7	23.20	7.88	17.18	24.00	12.82	49.72	30.77	383.15	8.85	61.85345
0.6	16.58	2.42	12.91	13.81	12.51	56.82	31.19	372.24	6.68	59.71049
0.5	12.46	8.43	12.99	16.38	12.80	24.63	32.50	383.15	2.52	79.77528
0.4	1.128	12.21	25.23	25.42	12.54	59.84	33.55	449.81	1.02	9.574468
0.3	1.11	6.89	12.71	20.06	12.96	56.86	31.25	450.89	1.0066	9.315315
0.2	1.10	6.52	15.93	16.38	12.57	25.17	34.17	420.48	1.002	8.909091
0.1	1.00	0.54	15.54	28.26	12.54	54.62	34.76	403.99	1	0

## Experiment with simulated data

**Figure 2:**
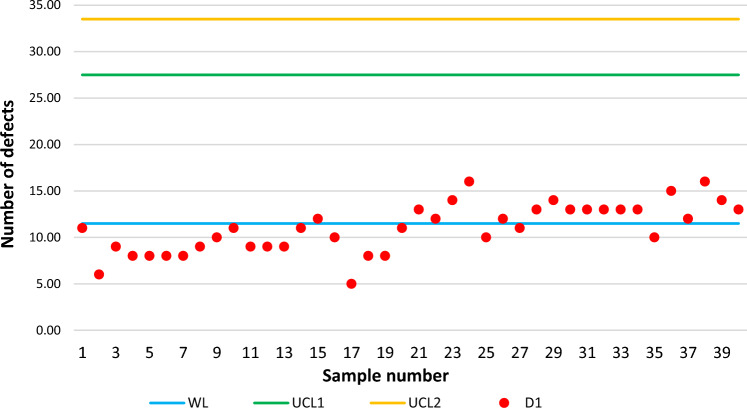
DS-Weibull scheme for the first stage simulated data.

In this section, the performance of the proposed control chart will be tested through simulated data using information from Refs.^14,18^ in the “Case study” section. Here, are taken 20 real samples with sample size $n=20$. Then they generated 20 simulated samples with $n=20$ also, where these last 20 samples belong to the process out-of-control. Here, ${p}_{0}=0.5810$ (proportion of defects in control) and ${p}_{1}=0.8244$ (proportion of defects out-of-control). In our case, we cannot use the first original samples because these belong to a constant sample size $n=20$. According to the below, we will use the ${p}_{0}$ and ${p}_{1}$ values to simulate the samples but using the two sample sizes corresponding to our control chart. From Ref.^14^ it is assumed that ${{\text{r}}}_{0}=200$, $\upbeta =1$ and ${\text{f}}=0.5$. With these conditions, from Table 1 we have the following variables: ${\text{WL}}= 11.50$, ${{\text{UCL}}}_{1}=27.50$, ${{\text{UCL}}}_{2}= 33.50$, ${{\text{n}}}_{1}=16$ and ${{\text{n}}}_{2}=28$. For the first 20 subgroups (the process in control) the following defects ${D}_{1}$ are generated using a binomial distribution with $p=0.5810$ and $n=16$ parameters: 11, 6, 9, 8, 8, 8, 8, 9, 10, 11, 9, 9, 9, 11, 12, 10, 5, 8, 8, 11. Then, the last 20 subgroups are generated when the process is out-of-control, $p=0.8244$: 12, 14, 16, 10, 12, 11, 13, 14, 13, 13, 13, 13, 13, 10, 15, 12, 16, 14, 13. Figure 2 shows the DS-Weibull scheme for this first stage.

From the previous scheme, it can be seen that subgroup number fifteen and subgroup seventeen are the two first subgroups where it is necessary to take a second sample. Thus, we must generate defects with binomial distribution and $p=0.8244$ and $n=28$.

**Figure 3:**
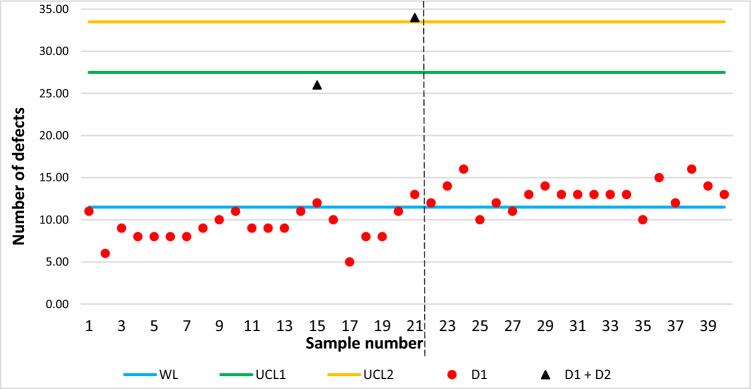
DS-Weibull scheme for the second stage simulated data.

Figure 3 shows the scheme, now with $({D}_{1}+{D}_{2})$ defects plotted.

Now in subgroup fifteen, $\left({D}_{1}+{D}_{2}\right)=26$, and the control chart continues, but in subgroup number twenty-one, $\left({D}_{1}+{D}_{2}\right)=34$, detecting the out-of-control signal. The dotted line indicates that at this point the control chart has detected the control output and corrective actions must be taken in the process. From Ref.^14^ it is known that 21–40 subgroups belong to the out-of-control state, so our proposed control chart detected the shift in the process just in the following subgroup from where the such change occurred, but Ref.^14^ detected the shift after of 6 subgroups.

**Figure 4:**
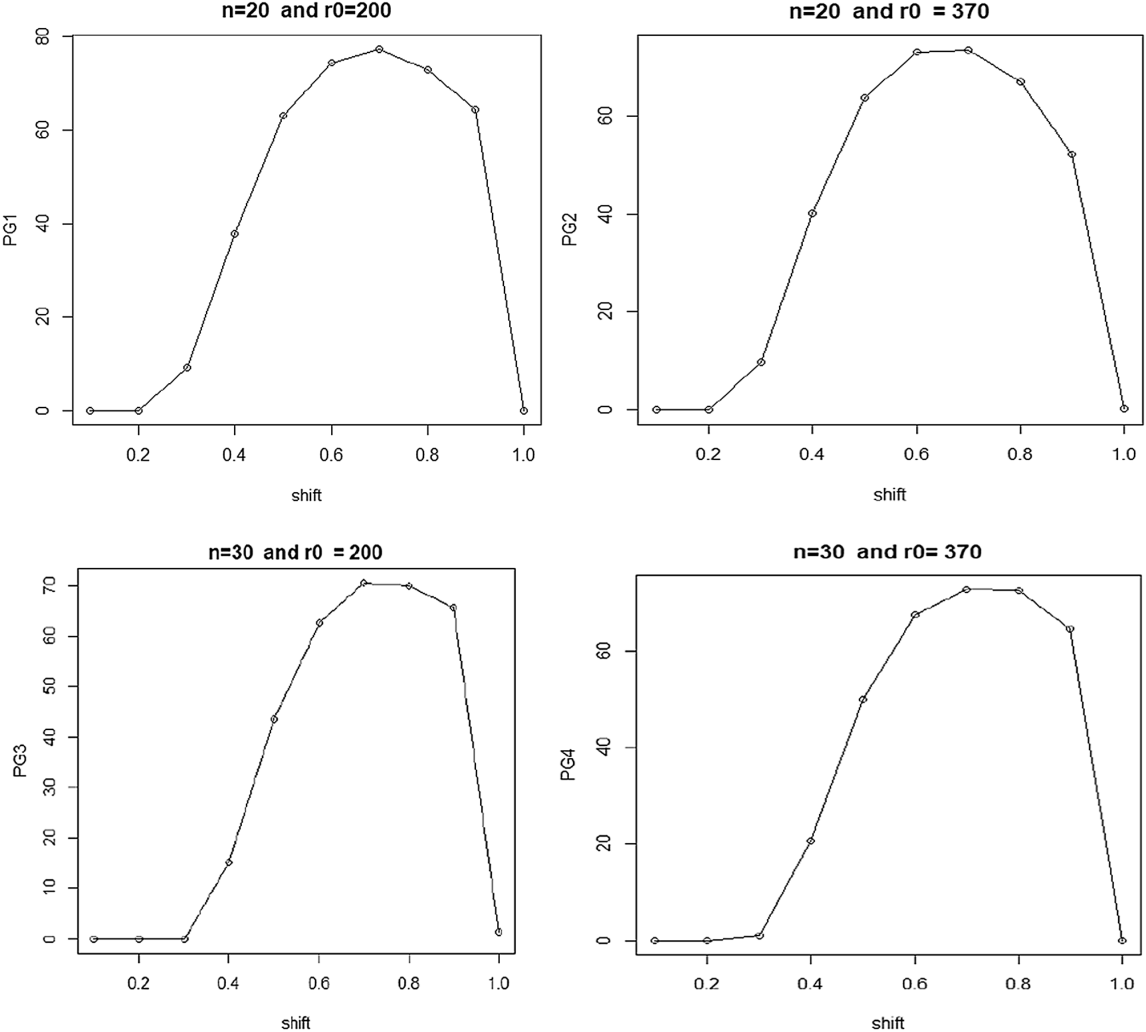
Percentage gains for various shifts and sample sizes, such as twenty and thirty.

In Fig. 4, the x-axis represents shifts in the process, and the y-axis displays percentage gains. Various schemes, including PG1, PG2, PG3, and PG4, are presented in the figure. PG1 (n = 20, r0 = 200), PG2 (n = 20, r0 = 370), PG3 (n = 30, r0 = 200), and PG4 (n = 30, r0 = 370) are depicted in the four figures. The same data is used in the table for all four schemes, with a preferable shift range of 0.40 to 0.90 for effective process control.

## Real life-example

In this section, a real-life example of strength measures in GPA for single fibers data in Table 7, as used by Ref. ^7^, is presented to illustrate the application of a two-stage sampling process with the Weibull distribution. The Weibull distribution is considered in the first stage, where 25 observations are used to establish the process as in control. The parameters are optimized and taken from Table 5, with the shape parameter set to 9.54 and the scale parameter set to 2.055. Subsequently, for the second stage sampling, Table 6 is utilized, and estimated parameters are obtained from 35 observations to further assess the process with optimized parameters, setting the shape as 14.54 and the scale as 2.69. The constraints outlined in the design of the control chart are to be followed from step 1 to step 3 accordingly.

**Table 7:** Data set of strength measure in GPA for single fibre (20-mm).

1.312	1.314	1.479	1.552	1.700	1.803	1.861	1.865	1.944	1.958
1.966	1.997	2.006	2.021	2.027	2.055	2.063	2.098	2.140	2.179
2.224	2.240	2.253	2.270	2.272	2.274	2.301	2.301	2.359	2.382
2.382	2.426	2.434	2.435	2.478	2.490	2.511	2.514	2.535	2.554
2.566	2.570	2.586	2.626	2.633	2.642	2.648	2.684	2.697	2.726
2.770	2.773	2.800	2.809	2.818	2.821	2.848	2.880	2.954	3.012

**Figure 5:**
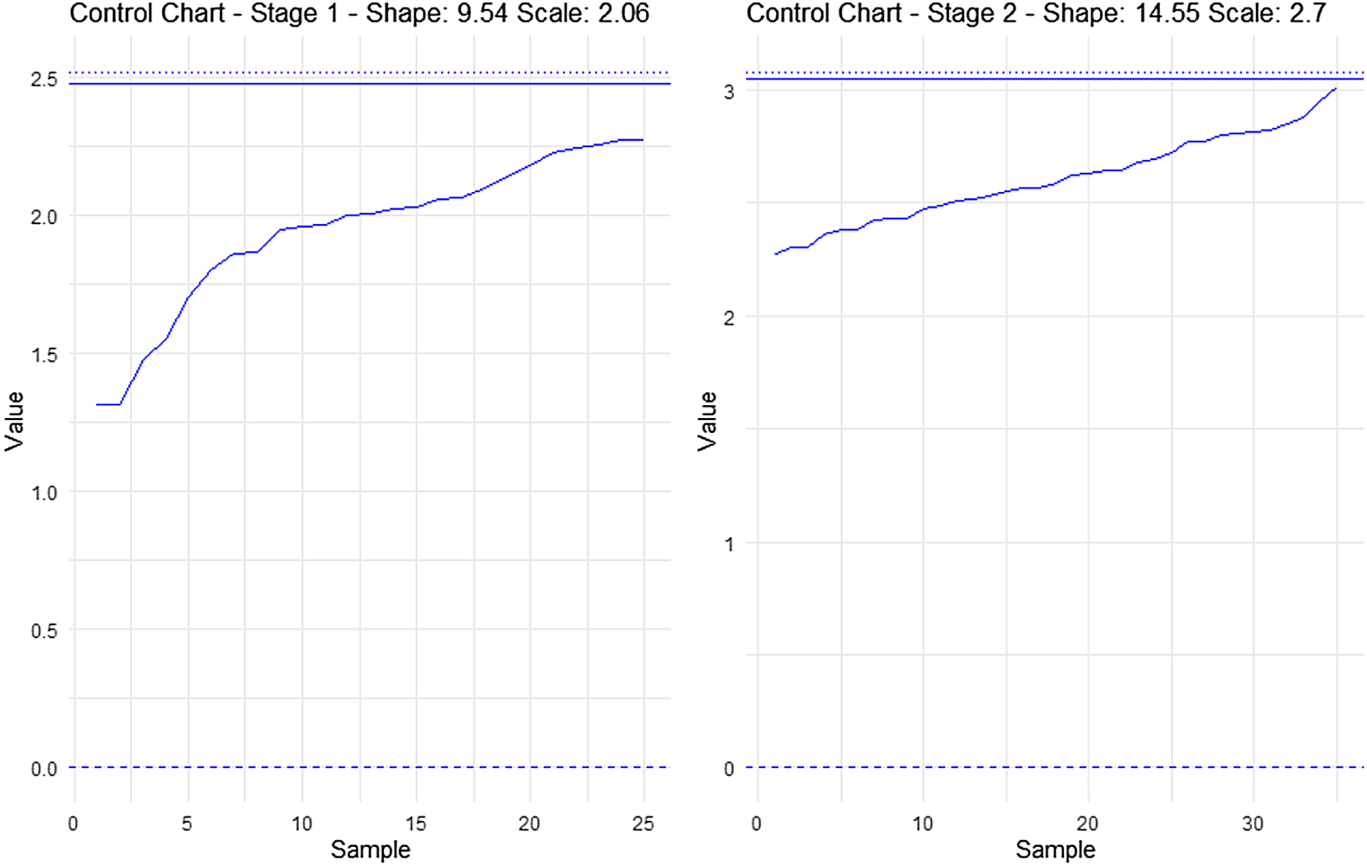
Control charts for sampling stage one and two using two different sets of optimized parameters.

**Figure 6:**
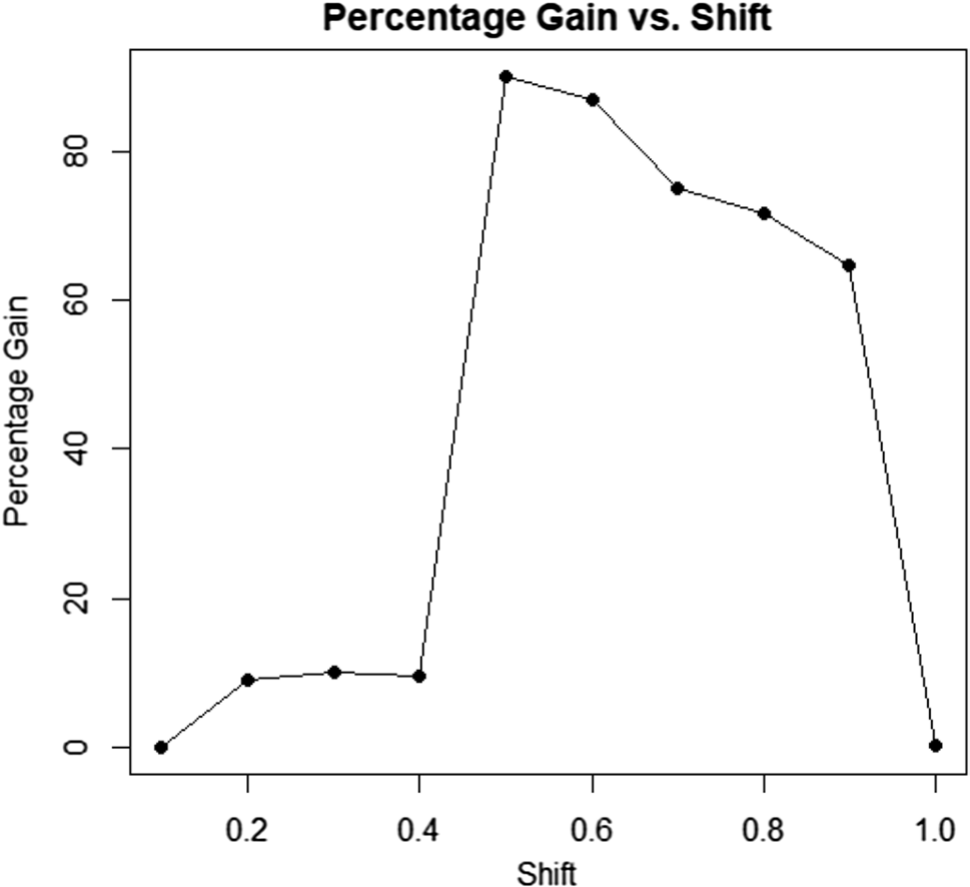
Using optimized parameter scheme for n = 25 and shape parameter 9.54 and scale as 2.055.

**Figure 7:**
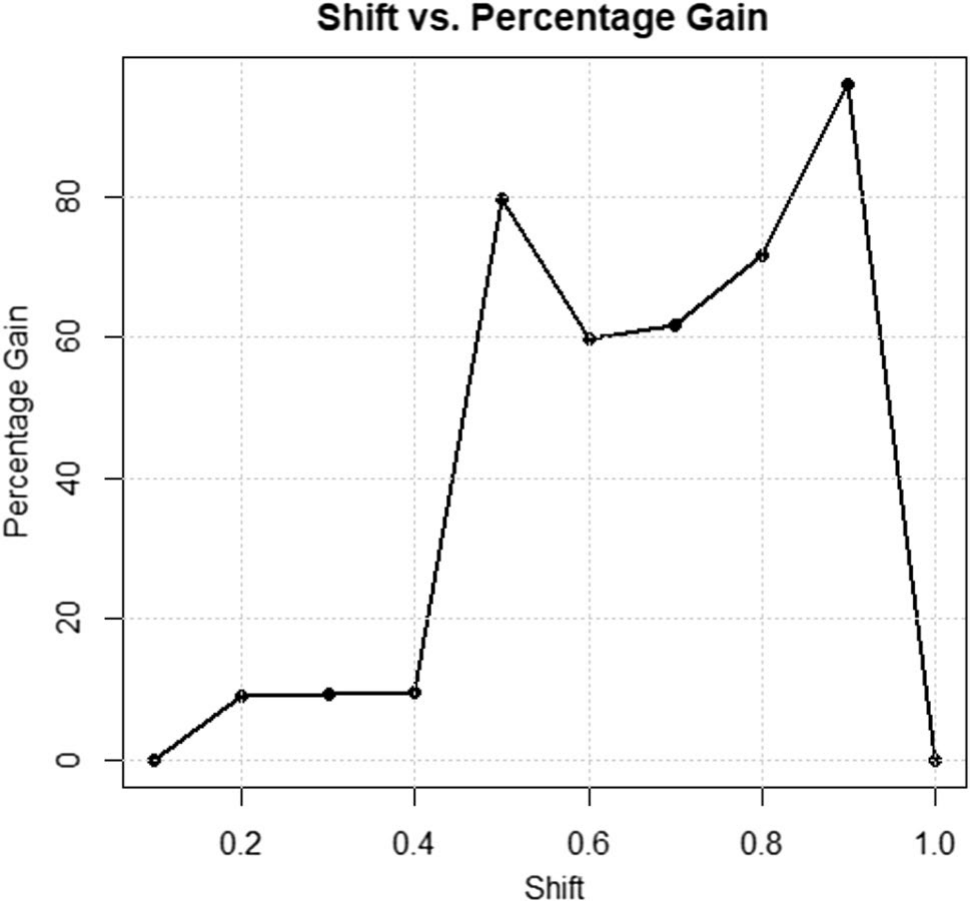
Using optimized parameter scheme for n = 35 and shape parameter 14.54 and scale as 2.69.

Figure 5 illustrates a control chart for the first sampling stage, depicting a process effectively in control, indicated by stable data points conforming to established constraints. The second graph extends the control chart to the second sampling stage, reaffirming process control established in stage one and reflecting continued stability with an additional forty observations. These control charts emphasize the reliability and stability of the process across both sampling stages, in line with the outlined constraints. Figure 6 shows increasing percentage gains with decreasing shift values (1 to 0.1), fitting a Weibull distribution (shape = 9.54, scale = 2.055). The optimized parameters provide insights into favorable outcomes and tail behavior for statistical analysis using a first-stage sample of n = 25, as displayed in Table 5. Figure 7 demonstrates behavior with optimized parameters for n = 35, shape = 14.54, and scale = 2.69 across different sample sizes. Analyzing shifts and percentage gains from 1 to 0.1 reveals crucial dynamics for SPC. The gains peak at a shift of 0.9 (95.89%), offering insights into system sensitivity and aiding in optimizing control parameters for process monitoring.

## Conclusions

Non-normal control charts are important to measure because they provide a way to monitor processes that produce data that is not normally distributed. These charts can help identify process shifts and trends that may not be detected with traditional control charts. Non-normal control charts can also help identify special causes of variation that may be present in the process. By monitoring these charts, organizations can take corrective action to improve process performance and reduce the risk of producing defective products. In this research work a new control chart is constructed for the detection of the lifetime of attribute control charts using Weibull distribution under truncated life test for double-stage sampling. The New control chart is much better based on average run length and average time to signal. The DS-Weibull chart shows better performance concerning $AR{L}_{1}$ metric for small and moderate change in the mean of the process when shift values are ranges $(f=0.4\text{ to }0.9 )$ compared with single sampling Weibull chart. The presented approach is assessed using both simulated and real-world datasets. A comparison with the conventional method reveals that the proposed control chart effectively operates with real-life data, demonstrating improved accuracy and efficiency in estimating the lifetime of the attribute control chart. This statistical method, involving two sampling stages, exhibits promise for future applications in biostatistics. It facilitates the continual monitoring and analysis of data over time, effectively identifying trends and patterns across various data types, such as patient outcomes, laboratory results, and quality control measures. The suggested control chart, coupled with cost analysis, can serve as a subject for future research. Additionally, exploring the application of the proposed control chart with repetitive sampling is a potential avenue for future investigation.

## Data Availability

The datasets used and/or analysed during the current study available from the corresponding author on reasonable request.
